# Phenotypic divergence despite low genetic differentiation in house sparrow populations

**DOI:** 10.1038/s41598-017-18718-8

**Published:** 2018-01-10

**Authors:** Shachar Ben Cohen, Roi Dor

**Affiliations:** School of Zoology, Faculty of Life Sciences, Tel Aviv University, Tel Aviv, Israel

## Abstract

Studying patterns of phenotypic variation among populations can shed light on the drivers of evolutionary processes. The house sparrow (*Passer domesticus*) is one of the world’s most ubiquitous bird species, as well as a successful invader. We investigated phenotypic variation in house sparrow populations across a climatic gradient and in relation to a possible scenario of an invasion. We measured variation in morphological, coloration, and behavioral traits (exploratory behavior and neophobia) and compared it to the neutral genetic variation. We found that sparrows were larger and darker in northern latitudes, in accordance with Bergmann’s and Gloger’s biogeographic rules. Morphology and behavior mostly differed between the southernmost populations and the other regions, supporting the possibility of an invasion. Genetic differentiation was low and diversity levels were similar across populations, indicating high gene flow. Nevertheless, the southernmost and northern populations differed genetically to some extent. Furthermore, genetic differentiation (*F*_ST_) was lower in comparison to phenotypic variation (*P*_ST_), indicating that the phenotypic variation is shaped by directional selection or by phenotypic plasticity. This study expands our knowledge on evolutionary mechanisms and biological invasions.

## Introduction

The study of phenotypic variation among natural populations and its relation to ecology is one of the central concepts in the field of evolutionary biology^1^. Identifying patterns of variation can provide insight into the forces driving evolutionary processes, whether natural selection, phenotypic plasticity, genetic drift – or an interplay between more than one of these processes^2,3^. Latitudinal variation in morphology and color is often explained by two important biogeographic rules^4,5^. Bergmann’s rule predicts that larger body size should be advantageous to homoeothermic animals in colder climates (usually found in northern latitudes), due to lower surface-to-volume ratio allowing better heat conservation^6,7^. However, there is disagreement on the validity of this rule for birds (42% vs. 72% of bird species^8,9^). Gloger’s rule predicts that animals should be more heavily pigmented in humid than in arid habitats, supported by adaptive explanations (matching between background color and feathers which serves as camouflage, or higher resistance of dark feathers to bacteria, which thrive more in humid habitats^10,11^). The empirical evidence for this rule is convincing, as 96% of bird species conform to this prediction^8^.

Population genetics tools are essential for understanding evolutionary mechanisms^12^. Neutral genetic differentiation (measured, for example, by *F*_ST_ index^13^) can indicate the level of gene flow between populations. In order to determine the evolutionary mechanism, genetic differentiation of quantitative traits (*Q*_ST_) is compared to the neutral genetic differentiation (*Q*_ST_-*F*_ST_ comparison). Together, they point to the main evolutionary scenario: directional selection (*Q*_ST_ > *F*_ST_), stabilizing selection (*Q*_ST_ < *F*_ST_), or genetic drift (*Q*_ST_ = *F*_ST_)^14^. *Q*_ST_ is difficult to measure in wild populations, since it requires rearing of animals in common garden conditions; therefore, the phenotypic divergence (*P*_ST_) is commonly used as a proxy of *Q*_ST_^15–17^.

Biological invasions threaten biodiversity and cause major economic losses worldwide^18,19^. Invasive species nevertheless provide an opportunity for studying evolutionary and ecological processes in real time^20^. Successful invaders must quickly adapt to novel environments and expand their range beyond the introduction site, eventually leading to phenotypic differentiation between populations^21^. For example, reduced levels of neophobia (the tendency to avoid novel objects or foods^22^) and increased exploratory behavior (searching behavior without immediate requirement^23^) may have an advantage in successful introductions^24^, and are therefore expected to characterize introduced populations^25,26^. However, it is not entirely understood how populations acquire such novel adjustments^27^. Reduction in genetic diversity is expected due to bottlenecks^28,29^, which may hinder adaptive evolution^30^. Furthermore, genetic evolution probably requires substantial time to operate^31^ (but see ref.^32^). Phenotypic plasticity, the ability of a genotype to express different phenotypes as a response to the environment, may therefore play an important role in the rapid habituation of introduced populations^33^.

The house sparrow (*Passer domesticus*) is one of the world’s most common bird species. Due to their commensal nature, and following numerous successful human-mediated introductions, house sparrows are abundant in almost every human-populated habitat^34^. Many well-documented introduction events exist around the world^34–36^. The exceptional success of the house sparrow as an invader, its great abundance, and the extensive knowledge on its biology and distribution, have made it a popular model in invasion biology^34,37^. One of the classic textbook study cases of rapid evolution in invasive populations was documented in house sparrows in North America^38,39^. A distinct pattern of variation in body size and color occurred within a time span of no longer than a century, corresponding to Bergmann’s and Gloger’s rules.

The house sparrow is native to Israel and abundant throughout the country. The local subspecies, *P*. *d*. *biblicus*, belongs to the Palearctic group^36^. However, in the southernmost part of Israel (Eilat area) sparrows appear to be smaller and brighter compared to other populations^40,41^. Thus, it was suggested that the southern populations may have been influenced by an Oriental subspecies, *P*. *d*. *Indicus*, introduced by ships via the Red Sea (which became possible from the 1950s, when the port of Eilat was established). At the same time, due to a steep climatic gradient in Israel from north to south^42^, a similar pattern of variation in both size and color is expected, in accordance with Bergmann’s and Gloger’s rules. Mean annual rainfall ranges between 25 mm in the south and 1,000 mm in the north, while mean annual temperature varies from 16 °C to 25 °C, respectively.

In contrast to other introductions of house sparrows, the assumed invasion in Israel is based on observational data alone and has not been quantitatively tested. In this study, we examined whether variation in morphology and color agrees with the expectations of the two mentioned biogeographic rules, and whether differentiation is greater between the southernmost region (Eilat) and other regions, in support of the invasion scenario. We measured behavioral variation between regions, since lower levels of neophobia and increased exploration are expected in the southernmost region, due to the potential advantage for invasive populations. We examined whether genetic structure occurs between populations, and searched for additional genetic “signatures” of invasion, such as reduced diversity. Finally, we examined whether the neutral genetic differentiation between populations, *F*_ST_, is significantly smaller than the differentiation of phenotypic traits, *P*_ST_, indicating local adaptation.

## Results

### Morphological Traits

**Figure 1:**
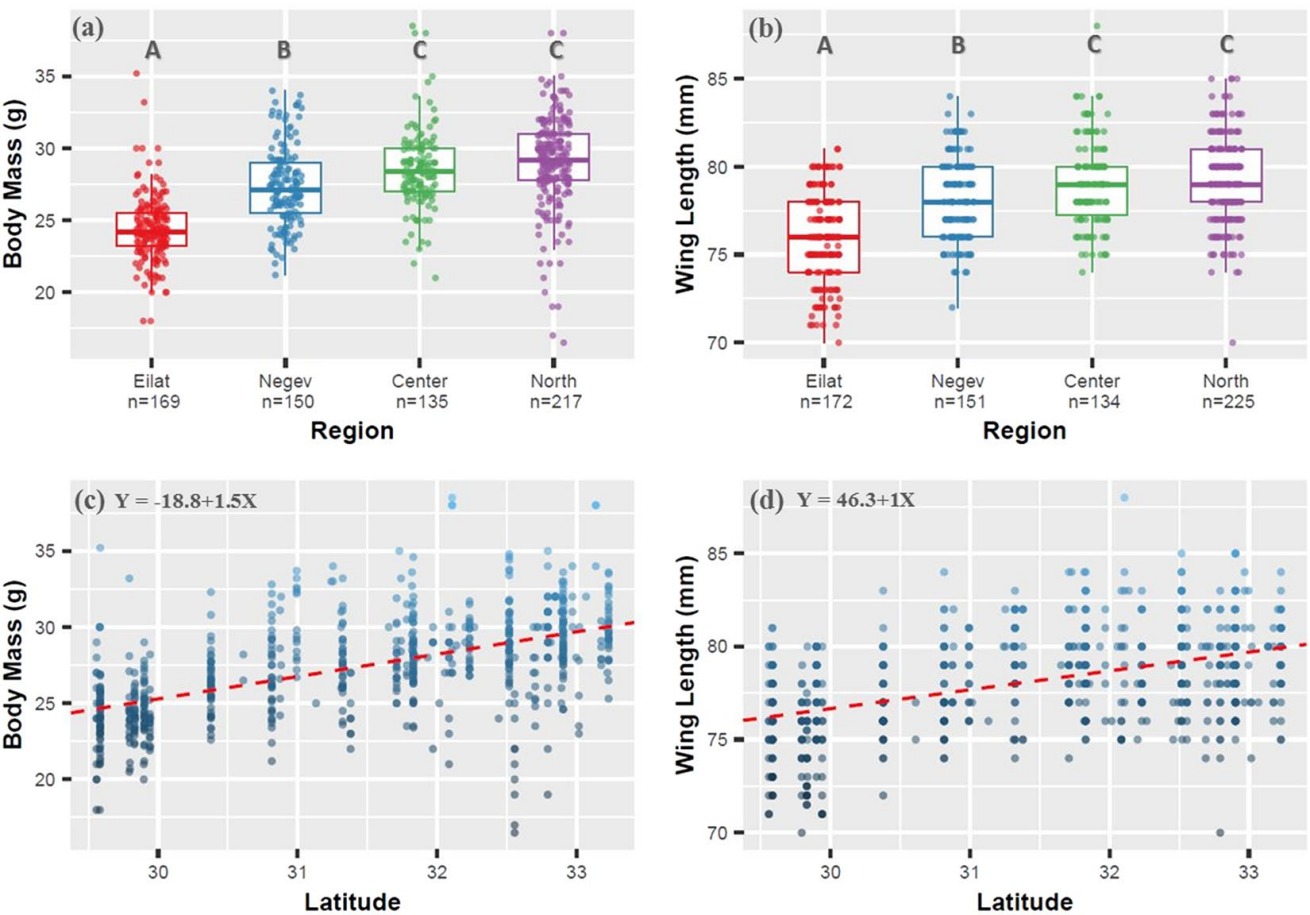
(**a**,**b**) Results of one-way ANOVA for two morphological traits, body mass and wing length. Heavy line within each box represents the sample median. Lower and upper limits of each box represent the 25% and 75% quartiles, respectively. Limits of vertical lines (whiskers) represent the min and max values, excluding outliers. Letters above boxes (A, B, C) represent significant differences in post-hoc analyses. Eilat (A) significantly differs from both the Negev (B) and Center, North (C), while the Center does not differ from the North. **(c**,**d)** Results of regression analyses of two morphological traits, body mass and wing length, plotted against latitude.

Five morphological traits were measured for sparrows from 18 populations and from museum specimens (N = 692; 398 males, 294 females; All mean values for each trait by region are listed in Supplementary Table S1). In one-way ANOVAs, four out of five traits differed among the four regions (from south to north: Eilat, Negev, Center, North) after Bonferroni correction for multiple comparisons: Body mass: *F*_3,667_ = 98, *P* < 0.001; Wing length: *F*_3,678_ = 65.5, *P* < 0.001 (Fig. 1a,b); Tarsus length: *F*_3,423_ = 4.98, *P* < 0.05; Bill length: *F*_3,661_ = 24.73, *P* < 0.001. Variation in bill width did not differ among regions (*F*_3,424_ = 2.21, *P* = 0.43). In pairwise post-hoc Tukey tests we found a strong indication for morphological divergence (in all traits but tarsus length) between the region of Eilat and the more northern regions. Individuals from the area of Eilat were morphologically similar to the Oriental subspecies (*P*. *d*. *indicus*) in body size measurements^36,43,44^.

We examined the contribution of latitude and climate features to the variation in morphology and color among populations. Precipitation and temperature PCAs were highly correlated with latitude (*r* = −0.93 for precipitation, *r* = 0.73 for temperature); therefore, in order to avoid the effects of multicollinearity, they were removed from following analyses. In a regression analysis with latitude as a predictor, all five traits showed a significant spatial pattern of becoming larger towards northern latitudes: Body Mass: *F*_1,669_ = 282.5, *r* ^2^ = 0.296, *P* < 0.001; Wing length: *F*_2,679_ = 95.8, *r* ^2^ = 0.218, *P* < 0.001 (Fig. 1c,d); Tarsus length: *F*_1,425_ = 13.9, *r* ^2^ = 0.029, *P* < 0.001; Bill Length: *F*_1,663_ = 58.05, *r* ^2^ = 0.079, *P* < 0.001; Bill width: *F*_1,426_ = 5.4, *r* ^2^ = 0.02, *P* = 0.01. For traits which were sexually dimorphic (wing length and bill width), we conducted separate ANOVA and regression tests (results are given in Supplementary Table S2).

### Color Brightness

Color brightness was analyzed for 380 males using a spectrophotometer on three parts of the body (belly, right and left cheeks). Mean values by region are listed in Supplementary Table S1.

**Figure 2:**
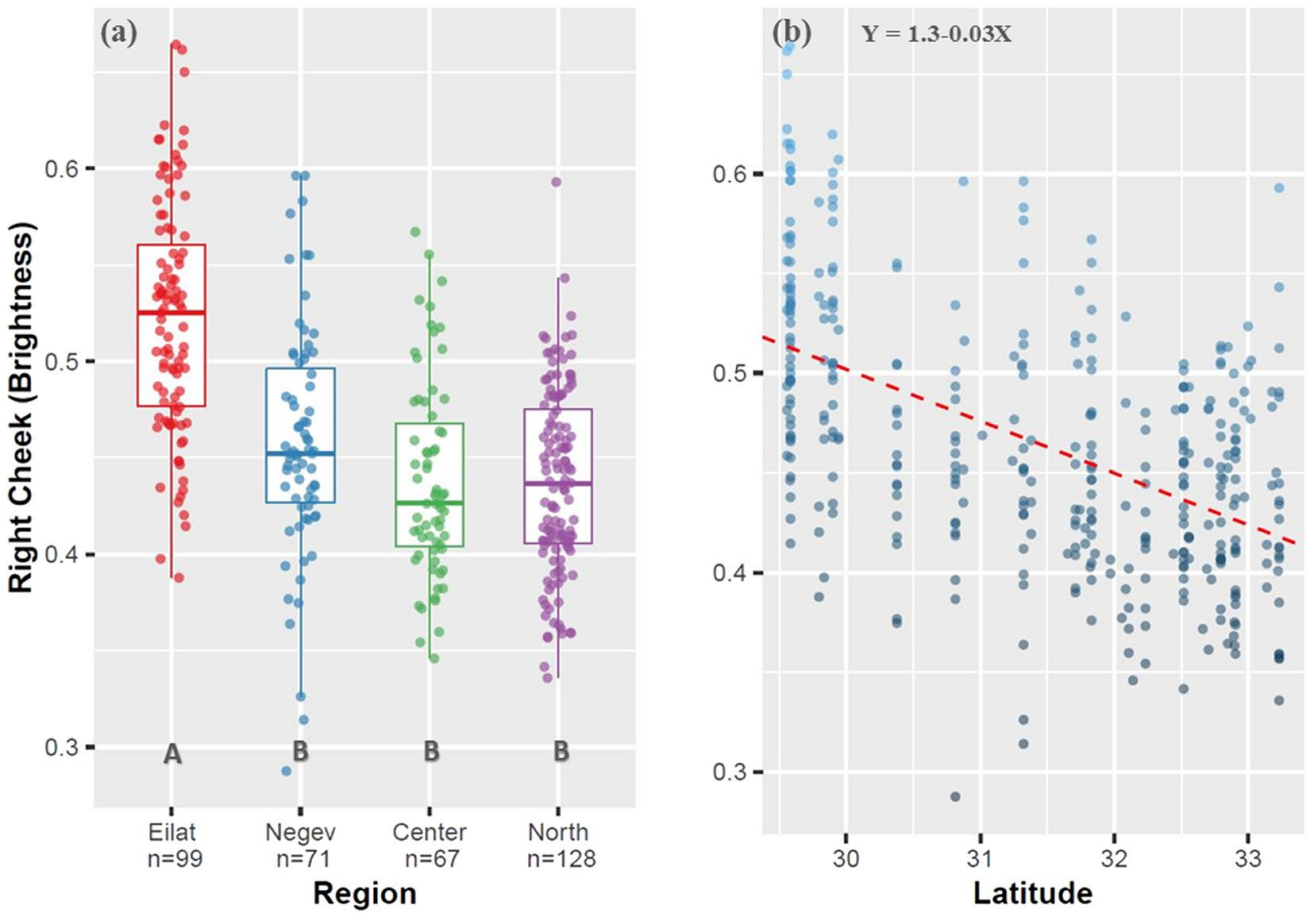
(**a**) Results of one-way ANOVA for right cheek brightness. Heavy line within each box represents the median. Limits of boxes represent the 25% and 75% quartiles, and limits of whiskers represent min and max values excluding outliers. Letters below boxes (A, B) represent significant differences in post-hoc analyses. Eilat (A) significantly differs from all other three regions, which are not different from one another **(b)** Results of regression analysis of right cheek brightness plotted against latitude.

All three color measurements differed between regions: right cheek brightness (*F*_3,361_ = 53.1, *P* < 0.001; Fig. 2a), left cheek (*F*_3,361_ = 51.6, *P* < 0.001), and belly (Welch’s ANOVA: *F*_3,186_ = 44.9, *P* < 0.001; Kruskal-Wallis χ^2^: 101.1, *P* < 0.001). In post-hoc analyses, the only significant difference in right cheek was between Eilat and the other three regions (*P* < 0.001). Left cheek differed between Eilat and the other regions (*P* < 0.001), and Center differed from the Negev region (*P* < 0.01). Belly brightness differed between Eilat and the North and Center (*P* < 0.001), but not from the Negev (*P* = 0.88), while the Negev differed from both the North and Center regions (*P* < 0.001). All three traits showed a significant spatial pattern of becoming darker towards the northern latitudes: right cheek (*F*_1,363_ = 130.9, *r* ^2^ = 0.263, *P* < 0.001; Fig. 2b), left cheek (*F*_1,363_ = 99.9, *r* ^2^ = 0.214, *P* < 0.001), belly brightness (*F*_1,365_ = 135.2, *r* ^2^ = 0.268, *P* < 0.001).

### Behavioral Assay

**Figure 3:**
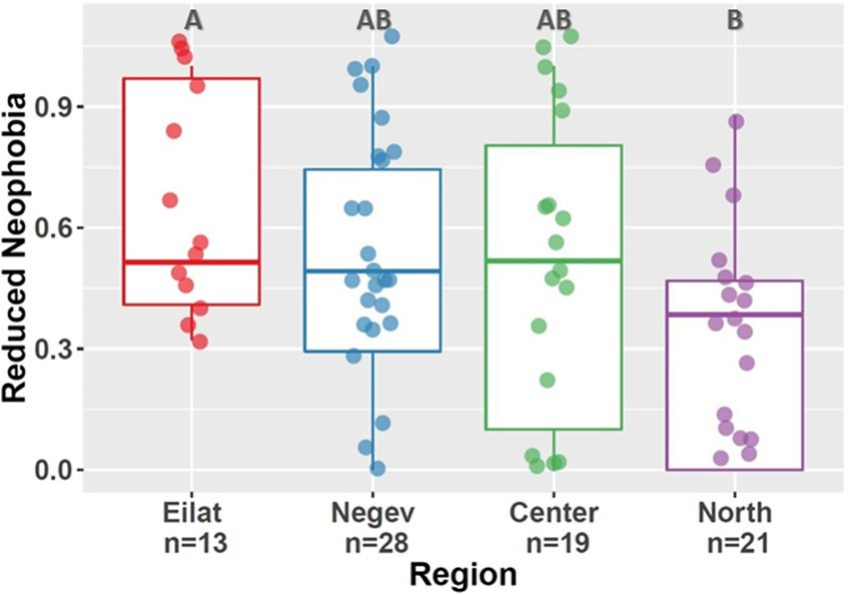
Variation in attributes of neophobia between regions. Higher score represents reduced neophobic response. Significant difference found in post-hoc Dunn’s Test between the region of Eilat and the North (represented by letters above boxes). Eilat (A) significantly differs from the North (B), but not from the Negev and Center (AB).

Behavioral assays of 81 male sparrows were scored for differences in exploratory behavior and neophobia between regions. Measures of exploratory behavior greatly varied among individuals (mean of hops ± SD: 44.6 ± 62.6; flight incidents: 8.7 ± 14.7; location changes: 13.7 ± 22.3; quarters visited: 2.1 ± 1.3; perches visited: 1.4 ± 1.8); however, we did not find significant variation among regions (χ^2^ = 0.69, df = 3, *P* = 0.87). Neophobia levels varied among regions (χ^2^ = 8.59, df = 3, *P* = 0.04; Fig. 3). Post-hoc analysis showed that the only significant pairwise difference was between Eilat and the North (*P* = 0.02). Sparrows in Eilat were less neophobic than those in the North.

### Genetic Analysis

We genotyped 267 sparrows (152 males, 115 females) from 14 populations, for 8 microsatellite loci. Overall, measures of genetic diversity did not differ among populations (Table 1). Expected heterozygosity (*H*_E_) was very similar in all 14 populations (0.74 – 0.79). Estimates of allelic (*A*_R_) and private allelic richness (*A*_PR_) were not lower in populations from the Eilat region than in all other populations. *F*_IS_ estimates were mostly close to zero, and did not differ among populations (95% CIs comparisons). Heterozygosity excess as an indication of recent bottlenecks was non-significant in all populations but one (Hulda).

**Table 1:** Summary statistics of genetic diversity attributes.

Population	N	*H*_E_	*F*_IS_	*A*_R_	*A*_PR_	Heterozygosity excess
Eilot	19	0.77	0.064	7.16	0.14	NS
Elifaz Samar	14	0.76	0.013	6.93	0.13	NS
Yotvata Grofit	28	0.79	0.088	7.21	0.21	NS
Faran	20	0.77	0.016	7.78	0.42	NS
Idan	19	0.77	0.113	7.09	0.08	NS
Tlalim	13	0.79	0.070	7.5	0.07	NS
Gilat	20	0.78	0.005	7.55	0.14	NS
Avigdor	15	0.77	0.048	7.09	0.02	NS
Maale Hahamisha Ramat Rachel	10	0.74	−0.013	6.88	0.14	NS
Hulda	20	0.79	0.090	7.43	0.24	0.02
Gaash	19	0.78	0.006	7.2	0.12	NS
Beit Alfa	20	0.74	0.001	7.39	0.09	NS
Ein Hamifratz	20	0.76	−0.004	7.65	0.35	NS
Dafna Amir	25	0.78	0.016	7.87	0.35	NS

N = individuals per population; *H*_E_ = expected heterozygosity; *F*_IS_ = inbreeding coefficient, high values indicate of high level of inbreeding; *A*_R,_ *A*_PR_ = allelic richness and private allelic richness, measured with HP-rare; Heterozygosity excess = results of 1-tail Wilcoxon rank test. Non-significant (NS), or the significant *P*-value for the population, measured with BOTTLENECK.

AMOVA revealed that most of the genetic variation (99.16%) was explained within populations, corresponding to a low global genetic differentiation over loci (*F*_ST_ = 0.0084). Sex-specific genetic differentiation was similar for females (*F*_ST_ = 0.012) and males (*F*_ST_ = 0.010). Clustering results also revealed a lack of genetic structure, suggesting K = 1 as the most likely cluster (out of 14). Although global tests revealed an absence of genetic structure, pairwise comparisons between populations gave some significant *F*_ST_ values, specifically between the southernmost population and the central and northern populations (Table 2). In support of these results, we also found a correlation between geographic distance and genetic differentiation among populations in a Mantel test (Z = 145.3, *r* = 0.37, *P* < 0.01), revealing a pattern of isolation by distance (IBD).

**Table 2:** Pairwise geographic and genetic distances between 14 populations.

	1 Eilot	2 Elifaz Samar	3 Yotvata Grofit	4 Faran	5 Idan	6 Tlalim	7 Gilat	8 Avigdor	9 Maale Hahamisha Ramat Rachel	10 Hulda	11 Gaash	12 Beit Alfa	13 Ein Hamifratz	14 Dafna Amir
1	—	23.7	36.3	89.9	139.8	157.3	195.1	236.6	248.3	249.1	294.0	328.3	368.4	409.5
2	0.0115	—	12.7	66.2	116.1	135.0	173.1	214.0	224.9	226.1	271.1	304.7	345.1	385.8
3	−0.0005	0.0015	—	53.7	103.5	124.3	162.6	202.9	212.9	214.6	259.6	292.4	333.2	373.4
4	0.0007	0.0032	0.0052	—	49.9	77.2	115.2	152.7	159.9	163.0	208.0	238.7	280.2	319.7
5	−0.0029	0.0065	−0.0028	0.0075	—	52.1	82.2	111.5	112.6	118.7	163.0	189.4	232.4	270.1
6	0.0066	−0.0046	0.0008	0.0104	0.0099	—	38.4	79.5	97.2	93.3	137.5	180.1	214.1	261.3
7	**0.0188**	0.0019	0.0054	0.0069	0.0092	0.0042	—	43.8	70.4	60.3	102.2	151.7	180.4	231.2
8	0.0103	0.0097	0.0055	0.0034	0.0038	0.0045	−0.0021	—	36.9	18.6	58.5	110.6	136.6	188.6
9	0.0151	0.0150	**0.0207**	0.0023	0.0061	**0.0272**	0.0169	0.0044	—	21.8	53.2	83.0	120.3	164.1
10	0.0117	0.0022	0.0049	0.0083	0.0039	0.0001	−0.0001	0.0014	0.0044	—	45.0	92.2	120.9	171.0
11	**0.0269**	0.0026	**0.0124**	**0.0154**	0.0068	0.0118	−0.0027	0.0069	**0.0238**	0.0064	—	65.2	78.8	134.5
12	**0.0283**	0.0071	**0.0164**	0.0041	**0.0150**	**0.0212**	0.0015	0.0041	**0.0199**	0.0106	0.0069	—	53.1	81.5
13	**0.0248**	−0.0043	**0.0124**	0.0094	0.0099	0.0122	−0.0008	0.0093	0.0159	0.0102	0.0026	−0.0008	—	62.4
14	**0.0245**	0.0069	**0.0121**	0.0066	**0.0128**	0.0066	−0.0056	0.0031	**0.0292**	0.0069	0.0034	−0.0002	−0.0004	—

Pairwise geographic distances between 14 populations are presented above the diagonal and genetic differentiation values (*F*_ST_) appear below. A total of 18 significant *F*_ST_ values are in bold. Nine out of 18 of the significant values are between populations from the region of Eilat (Eilot, Elifaz-Samar, Yotvata-Grofit) and populations from the Center and North regions.

### Comparison of Phenotypic and Genetic Differentiation (*P*_ST_ - *F*_ST_)

Lower 95% CI values of phenotypic differentiation among populations, *P*_ST_, were compared to the upper 95% CI of neutral genetic differentiation^17^ (0.016 for males and females; 0.024 for males only). *P*_ST_ surpassed the global *F*_ST_ value for six out of eight phenotypic traits (Table 3). The only morphological traits for which *P*_ST_ was not larger than *F*_ST_ were bill width, which also did not show significant variation between regions, and left cheek brightness. Critical values of *c/h* ^2^ were therefore estimated for all traits but bill width and left cheek brightness, with lower values indicating greater robustness for the comparison between phenotypic and genetic differentiation^17^. Tarsus length, followed by body mass, had the smallest critical *c/h* ^2^ and largest *P*_ST_ value, while right cheek brightness had the largest critical *c/h* ^2^ value.

**Table 3:** Summary of comparisons between phenotypic and genetic differentiation (*P*_ST_-*F*_ST_) among house sparrow populations in Israel.

Trait	*P*_ST_	Lower 95% CI	Upper 95% CI	Critical *c/h* ^2^	*P*_ST_-*F*_ST_
Body mass	0.313	0.176	0.539	0.079	*P*_ST_ ≫ *F*_ST_
Wing length	0.253	0.109	0.445	0.131	*P*_ST_ > *F*_ST_
Tarsus length	0.443	0.292	0.680	0.046	*P*_ST_ ≫ *F*_ST_
Bill length	0.130	0.054	0.296	0.171	*P*_ST_ > *F*_ST_
Bill width	0.000	0.000	0.118	—	*P*_ST_ = *F*_ST_
Right cheek brightness	0.113	0.025	0.288	0.959	*P*_ST_ > *F*_ST_
Left cheek brightness	0.130	0.009	0.352	—	*P*_ST_ = *F*_ST_
Belly brightness	0.379	0.204	0.662	0.124	*P*_ST_ ≫ *F*_ST_

*P*_ST_ value estimated for *c* = *h* ^2^. Critical *c* = *h* ^*2*^ calculated only for traits whose upper *F*_ST_ CI > lower *P*_ST_ CI. Upper *F*_ST_ CI value used for the five morphological traits was 0.016 (males and females), and 0.024 for color brightness trait (male-only *F*_ST_).

## Discussion

We examined patterns of phenotypic variation in morphology, plumage coloration, and behavior in house sparrows and compared them to patterns of neutral genetic variation. Our two main motivations were: first, to understand the evolutionary processes driving the phenotypic variation between populations; and second, to investigate the hypothesis that introduction of sparrows had occurred in the southernmost part of our research area.

We found evidence for spatial patterns of variation in morphology and color, which correspond to a climatic gradient and also support the possibility of introduction. Furthermore, inter-population behavioral differences present a pattern fitting an invasion scenario. Genetic differentiation and diversity analyses revealed that gene flow occurs between populations. Comparison of the phenotypic variation to genetic differentiation indicated that the variation cannot be explained by genetic drift alone. Overall, the results indicate that the observed variation between phenotypic traits derives from a process of local adaptation or phenotypic plasticity, and is in congruence with studies of house sparrow populations in both native and introduced ranges worldwide^45–47^.

Variation in all morphological measures coincides with the prediction of Bergmann’s rule, as sparrows become larger towards the northern localities. Although evidence for the validity of Bergmann’s rule across species and its adaptive advantage are equivocal, it is considered one of the central biogeographic principles. Thus, our results indicate a possible selective pressure leading to local adaptation. Additionally, four out of five measurements revealed significant differentiation among regions, with emphasized divergence between the southernmost populations and the other, more northern, regions. This provides empirical support for the assumption that foreign populations of a different, smaller subspecies (i.e, *P*. *d*. *indicus*), may occur in the Eilat region.

Interestingly, out of the five morphological traits, bill width and tarsus length demonstrated the weakest pattern of variation in relation to latitude and between regions. Both are skeletal measurements, and thus are less prone to environmental changes (however they may be affected by ontogenetic plasticity). In contrast, body mass, wing length, and bill length of house sparrows have been shown to respond to seasonal changes^48,49^, fluctuate between breeding and non-breeding seasons^50^, and body mass can vary on a diurnal basis^46^. This pattern may be indicative of a substantial plastic effect in shaping the morphological variation.

An increase in coloration along the north-south gradient was also evident, fitting the expectations of Gloger’s rule. Presuming an adaptive advantage for Gloger’s rule, the results indicate an adaptive basis for variation in plumage coloration of house sparrows. Complementary to variation in body size, differentiation between regions in color was also most significant between Eilat and the rest of the localities, with sparrows from Eilat being brighter, resembling the Oriental subspecies^36^.

Variation in exploratory behavior was evident among individuals, but we could not detect significant differences among regions. Conversely, significant but weak differences were detected in neophobic response between Eilat and the North region. Individuals from Eilat were less averse to novelty (visited decorated perches more) and spent more time in the center of the arena, as opposed to individuals from the North. Assuming that foreign populations occur in the southernmost region, this finding agrees with the hypothesis that assumes an advantage to being less neophobic in a novel habitat. The lack of difference in exploratory behavior among regions could perhaps be explained by the “adaptive flexibility hypothesis”^25^, according to which proxies of behavioral flexibility are expected only in the initial stage of introduction. Furthermore, exploratory behavior and reduced neophobia may induce costs, such as the risk of being revealed to predators or ingesting poisonous foods^22^. Consequently, cautiousness is likely to be favored once populations have become established.

Genetic differentiation between populations was low, demonstrating a lack of structure, indicating high gene flow among populations. We did find a pattern of isolation by distance, as a substantial portion of the significant pairwise differentiation values were between the southernmost populations, and some of the populations in the north and center. This agrees with the invasion scenario, as populations influenced by foreign immigrants are expected to genetically differ from their native counterparts. However, due to strong commensalism and the extremely sedentary nature of sparrows^41,51^, dispersal could be linked to human settlements, which are sparser in the southern, arid, area of Israel compared to the rest of the country. This alone could be a cause for some of the divergence found, independent of the introduction scenario. In contrast to the invasion scenario expectation, we found no evidence of a recent bottleneck^52^. However, this may be due to an admixture with the local subspecies^53^ or by a quick recovery from the bottleneck^54^. Altogether, inferring whether an introduction event occurred based on genetic diversity alone is at best partial, although it can provide additional support for more concrete data.

Most of the variation in phenotypic traits was greater than the neutral genetic differentiation (*P*_ST_ > *F*_ST_), suggesting that phenotypic variation cannot be explained by genetic drift alone, but at least partially derives from natural selection. However, since the validity of the approximation of *Q*_ST_ by *P*_ST_ has been largely criticized^55^, the results should be interpreted cautiously. Contrary to *Q*_ST_, which measures additive genetic differentiation under common garden conditions^56^, *P*_ST_ is calculated from phenotypic data alone, and thus it cannot distinguish between the contribution of plasticity and genetic evolution to the observed variation.

Our analyses of the genetic differentiation are based on a dataset of eight microsatellite loci. Although many studies have used comparable number of markers (6–14 microsatellite) to examine genetic differentiation and for *F*_ST_ - *P*_ST_ comparisons^45–47,54,57–59^, more markers (SNPs) may provide a better estimate for the genetic differentiation^60^. Increasing the number of markers would probably decrease the confidence limit, but will have lesser effect on the estimate. In support, a panel of 6736 variable SNPs and a panel of 14 microsatellites produced very similar *F*_ST_ values in a study of house sparrow populations in Norway. However, the 95% confidence limit intervals for the microsatellite markers were wider^61^. That said, increasing the number of markers used may generate more reliable estimates of the genetic differentiation and effect our estimation of the level of adaptive evolution.

Since most of the documented phenotypic traits are plausibly affected by environmental conditions, some variation may be due to phenotypic plasticity. On the other hand, it is safe to assume that a genetic basis does exist for these morphological and color traits, as most of the discussed traits are heritable in house sparrows and other species^62–64^. Tarsus length, which is the least likely to be affected by phenotypic plasticity, had the smallest *c/h* ^2^ value, indicating that robustness of the *P*_ST_ - *F*_ST_ comparison was strongest for this trait.

Studying trait variation in a common species, such as the house sparrow, provides an excellent opportunity to investigate evolutionary and ecological questions. Our findings show that phenotypic variation over a climatic and latitudinal gradient agrees with recognized biogeographic rules, while also fitting the scenario of a possible introduction of foreign populations into the studied area. In order to further investigate the possibility of invasion, it may be informative to obtain genetic data from the original range of the subspecies in question, *P*. *d*. *indicus*. Comparing the low neutral genetic differentiation to the phenotypic variation indicates that random evolution by genetic drift is probably not the prime cause of the observed variation. Further investigation, which may include rearing of individuals from distant populations in common garden conditions, should determine whether variation has been shaped by a process of selection or by phenotypic plasticity. House sparrows have been experiencing severe population declines in vast parts of the world in recent decades^65,66^. Thus, expanding our current knowledge of its evolution and ecology may prove to be important for future conservation efforts for this ubiquitous and endearing bird.

## Materials and Methods

### Capture Sites and Sampling Procedures

**Figure 4:**
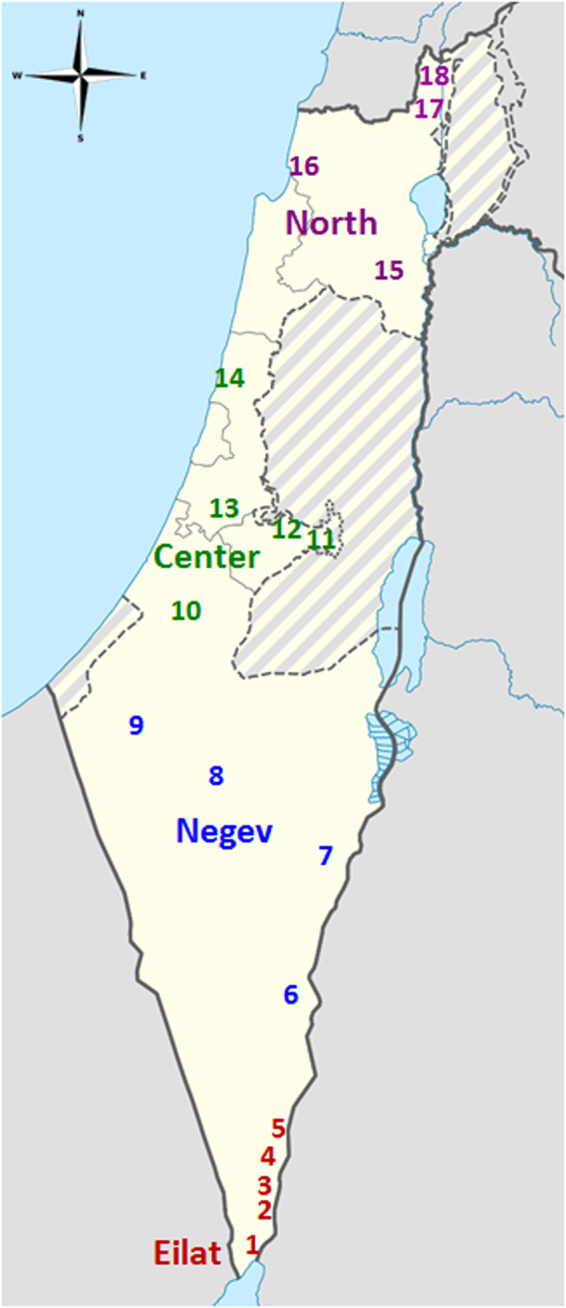
Map of sampling localities across Israel.

A total of 486 adult house sparrows (260 males, 226 females) were caught using mist nets in 18 localities during 4/2013, and between 11/2014 and 6/2015, from north to south of Israel (~420 km). All capture sites were located near livestock farms in suburban and rural settlements (Table 4, Fig. 4). All birds were released back at the capture site after sampling. Following capture, we measured body mass with a digital scale (0.01 g accuracy), wing length with a ruler (1 mm), tarsus length, bill length and width with a digital caliper (0.01 mm). A small blood sample (20-50 µl) was collected from the brachial vein and stored in a 1.5 ml tube containing blood lysis buffer^67^ (0.1 M Tris pH 8; 0.1 M Ethylenediaminetetraacetic acid (EDTA); 0.01 M NaCl; 0.5% sodium dodecyl sulphate (SDS)). All males were measured using a spectrophotometer (i1Pro X-rite, Grand Rapids, Michigan; aperture of 4.5 mm diameter) for analysis of color brightness, on three parts of the body: belly (bright colored feathers under the bib), and right and left cheeks (white/grey colored area beneath the eye). Color data were obtained for males only, since plumage variation is expected to be more evident in males. GretagMacbeth’s Eye-One Share v1.4 software was used to translate the data to a 380–730 nm spectrum table (10 nm intervals) and converted to RGB values using a spectral calculator spreadsheet (© 2001–2016 Bruce Justin Lindbloom, http://www.brucelindbloom.com). We analyzed variation in one component of RGB (green), indicative of the relative brightness of the sample, transformed from a 0–255 scale to a scale of 0–1, where 0 = black (dark), 1 = white (bright). We also measured morphology and color traits in specimens collected over the past 80 years in Israel and vouchered at the Steinhardt Museum of Natural History, Tel Aviv University (SMNHTAU; 138 males and 68 females). Museum specimens were not included in the genetic analysis.

**Table 4:** List of localities, coordinates and dates of sampling.

	Location	Region	Type	Date	Latitude (N)	Longitude (E)	Altitude (m)
1	Eilot	Eilat	Petting zoo	3/3/2015	29.5835	34.9604	89
2	Elifaz	Eilat	Dairy farm	17/4/2013	29.7928	35.0106	121
3	Samar	Eilat	Dairy farm	16/4/2013	29.8294	35.0238	92
4	Yotvata	Eilat	Dairy farm	2/3/2015	29.8995	35.0596	86
5	Grofit	Eilat	Dairy farm	15/4/2013	29.9393	35.0635	141
6	Faran	Negev	Dairy farm	4/3/2015	30.3776	35.1503	94
7	Idan	Negev	Dairy farm	22/2/2015	30.8140	35.2768	−174
8	Tlalim	Negev	Dairy farm	23/2/2015	30.9929	34.7735	364
9	Gilat	Negev	Dairy farm	15/6/2015	31.3225	34.6507	138
10	Avigdor	Center	Dairy farm	9/11/2014	31.7094	34.7439	65
11	Ramat Rachel	Center	Petting zoo	26/3/2015	31.7410	35.2165	809
12	Ma’ale Hahamisha	Center	Dairy farm	28/12/2014	31.8197	35.1115	805
13	Hulda	Center	Horse stable	28/1/2015	31.8293	34.8818	121
14	Gaash	Center	Petting zoo	2/1/2015	32.2323	34.8252	23
15	Beit Alfa	North	Dairy farm	5/4/2015	32.5171	35.4315	−85
16	Ein Hamifratz	North	Dairy farm	11/1/2015	32.9040	35.0972	6
17	Amir	North	Dairy farm	6/4/2015	33.1770	35.6236	76
18	Dafna	North	Dairy farm	6/4/2015	33.2299	35.6421	141

Locality numbers correspond to the numbers presented on the map in Fig. 4.

### Behavioral Assay

A total of 94 males (2–9 at each locality, mean ± SD: 6.6 ± 2.2) were randomly selected for a novel environment test during 2014–2015. In order to reduce stress caused by handling^68^, measurements and blood samples were taken after the assay was completed for each bird. Variation in exploratory behavior is generally assessed by observing animals in an artificial arena, while neophobia is measured by the response to novel items or foods^22^. The novel environment setting and assay we used is based on a classic “open field” test, adapted from previous protocols^69–71^. The arena was located in a standard camping tent (3.0 m × 2.40 m, 1.90 m height). The tent floor was divided into four quadrats and six artificial wooden perches were placed inside (1.0 m height, 50 cm “branch”). In order to stimulate a novelty response, three perches were decorated with “novel” objects, presumed foreign to the natural habitat of the sparrows (see Supplementary Figure S4 for pictures of the tent and the novel items). In order to avoid possible behavioral variation caused by social context^72^, sparrows were released into the tent separately, and left to freely explore the arena for approximately 15 minutes, filmed throughout with a GoPro Hero3 + camera. Videos were later analyzed, blind to the capture localities. Out of the 94 males we were able to analyze only 81 due to technical problems (seven assays were removed since no camera recording was available; six others were removed due to strong wind conditions).

Exploratory behavior was measured according to the following variables, summed to a final score: (1) Proportion of the tent explored – number of quarters visited (0 to 4), and number of perches used (from 0 to 6); (2) Location changes (each movement between quarters/ from quarter to perch/ from perch to perch); (3) Number of hops; (4) Number of flight incidents.

Each variable received a score, normalized for the duration of each assay, and relative to the best result. For example, if a bird visited 6 perches during 10 minutes received the best result for this variable ( = 1), then a bird that visited only 3 perches during 20 minutes would receive a score of 0.25 out of 1. A separate score was given for neophobic behavior, measured according to the proportion of decorated perches visited (out of total time perched), and the proportion of time spent in the center of the arena as opposed to its corners.

### Molecular Lab Procedures and Genotyping

Genomic DNA was extracted from blood samples of 267 sparrows captured in the field (152 males, 115 females; up to 20 representatives from each locality), according to the protocol of QIAGEN DNeasy blood & Tissue kit. DNA was amplified by polymerase chain reaction (PCR) for ten nuclear microsatellite markers: Pdo1^73^, Pdo5^74^, Pdo8^75^, CAM01, CAM02, CAM05, CAM10, CAM15, CAM17, CAM20^76^. Forward primers were labeled with either 6-FAM, ROX, VIC or NED fluorescent dyes and PCR reactions were performed separately for each marker. PCR Products were combined into three multiplex mixes for genotyping (see Supplementary Table S3), and allele size scoring of the results was performed with GeneMarker v2.6.7 (SoftGenetics, LLC), verified and amended by eye.

#### Analysis of genetic data

Occurrences of linkage disequilibrium (LD) and deviation from Hardy-Weinberg equilibrium (HWE) were tested for each population, using Arlequin v3.5.2.2^77^, as well as the presence of null alleles (checked with Cervus v3.0.7^78^). Markers Pdo8 and CAM20 were excluded from the analysis due to significant deviation from HWE and high null allele percentage (37.6% for Pdo8, 20% for CAM20). Alleles per locus ranged from 4 to 28 for the eight remaining markers. Since sample sizes in some localities were too small to obtain meaningful results, they were merged with the closest geographic locality (between 4 to 12 km distance between two merged populations), forming a total of 14 genetic populations. Genetic structure of populations was evaluated using two methods: first, analysis of molecular variance, AMOVA^79^, implemented in Arlequin. This is a hierarchical analysis that partitions total variance into covariance components due to intra and inter-individual differences and inter-population differences. Populations were allocated into pre-defined groups (Eilat, Negev, Center, North). The second method used was Bayesian clustering implemented in STRUCTURE v2.3.4^80^. STRUCTURE assigns samples into clusters (populations) using an admixture model, assuming correlation of allele frequencies, without prior knowledge of sample locality. We ran the clustering with a 20,000 burn-in period followed by 50,000 MCMC iterations for possible *K* = 1–14 populations, 10 times for each run (K). In order to infer which K was most likely, we ran the results in Structure Harvester^81^.

Genetic differentiation between populations was assessed by the *F*_ST_ index^13^, estimated using Weir & Cockerham’s *θ* ^82^, and implemented in Arlequin. Pairwise *F*_ST_ values were used to assess whether a pattern of isolation-by-distance (IBD) exists between populations, using the Isolation by Distance Web Service v3.23^83^. This application implements Mantel Test^84^ with 10,000 randomizations, for matrix correlation between genetic distance (*F*_ST_ values) and geographic distance (in km, determined using Coordinate Distance Calculator; http://www.boulter.com/gps/distance).

Levels of genetic diversity within populations were estimated by allelic richness (A_R_) and private allelic richness (A_PR_) in HP-Rare v1.1^85^, compensating for differences in sample size. Variation in expected heterozygosity (*H*_E_) and *F*_IS_ index^86^ were measured with the R package DiveRsity^87^. Detection of possible recent bottlenecks was achieved by measuring levels of heterozygosity excess, with BOTTLENECK v1.2.02^88^. We ran the software for Wilcoxon sign-rank test and mode-shift indicator^89^ with 1,000 replications under the two-phase model (T.P.M).

### Statistical Analysis

All statistical analyses, unless noted otherwise, were performed in R v3.1.2 (R Development Core Team 2014).

#### Variation of phenotypic traits between regions

The sampled populations were separated into four regions (Eilat, Negev, Center, North). Variation in morphology, color, and behavior among regions was estimated for each trait separately using Analysis of Variance (ANOVA) and post-hoc Tukey tests. For traits that deviated from assumptions of normality and homoscedasticity we used two alternatives, Welch’s ANOVA, and a Kruskal-Wallis test, followed by Dunn’s post-hoc test.

#### Spatial morphological trends

We used linear regression analyses in order to examine the factors contributing to the variation in morphology and color (separately for each trait) with latitude and climate as predictor variables. For traits that deviated from the model’s normality assumption we applied square-root transformations.

Exact GPS coordinates (latitude, longitude and altitude) were obtained for all localities and approximated for museum specimens using GPS-coordinates website (© 2016 http://www.gps-coordinates.net). Arc GIS v10.3 (Esri Inc.) was used to produce climatic layers for all coordinates, extracted from the WorldClim^90^ database (www.worldclim.org). We ran two separate PCAs for temperature and precipitation (Temperature PC1 and Precipitation PC1, explaining 79% and 99.7% of the variation, respectively).

#### Phenotypic variation vs. genetic differentiation

Divergence of phenotypic traits between populations, *P*_ST_, was compared to the neutral genetic differentiation, *F*_ST_. Global *F*_ST_ values and their 95% confidence intervals for males + females and for each sex separately were estimated with Arlequin.

1 $${P}_{{\rm{ST}}}=\frac{\frac{c}{{h}^{2}}{\sigma }_{B}^{2}}{\frac{c}{{h}^{2}}{\sigma }_{B}^{2}+2{\sigma }_{W}^{2}}$$

The value of *P*_ST_, defined in eq. 1, was estimated for each morphological and coloration trait assuming that *c* = *h* ^2^, representing the ratio between variation caused by additive genetic effects (*c*), and heritability (*h* ^2^). Phenotypic variance between (σ^2^_B_) and within (σ^2^_W_) populations was estimated using the MCMCglmm package in R^91^. For this analysis we used a subset of the color and morphology dataset, including only individuals for which we had genetic data as well. We constructed linear mixed models for all phenotypic traits, using population (locality) as a random effect and sex as a fixed effect for morphological traits, and year of sampling as a fixed effect for color traits. The default priors of the R package were used (65,000 bootstrap iterations, 15,000 burn-in period, 50 = sampling interval). To account for the robustness of the comparison, for each trait where *P*_ST_ exceeded *F*_ST_ we calculated the critical *c/h* ^2^ value, for which the upper 95% CI of *F*_ST_ equals the lower 95% CI of *P*_ST_^17^.

### Ethical Statement

All methods were carried out in accordance with the guidelines and regulations of the Israeli animal welfare law and the Israeli wildlife protection law. All capture and handling of birds, as well as all experiment protocols were approved and performed under permit (#L-14-062) granted by the Veterinary Service Center at the Sackler Faculty of Medicine, Tel Aviv University, according to the Israeli animal welfare law. The house sparrow is classified as a pest species under the Israeli wildlife protection law; therefore its capture for research purposes does not require an additional permit.

### Data Availability

The datasets generated and analyzed during the current study are available from the corresponding author upon reasonable request.

## Electronic supplementary material

Supplementary materials
