# Supplementary material for: Phenotypic divergence despite low genetic differentiation in house sparrow populations
Source: Sci Rep. 2018 Jan 10;8:394. doi: 10.1038/s41598-017-18718-8 (PMC5762629; doi:10.1038/s41598-017-18718-8)

Supplementary materials for:

Phenotypic divergence despite low genetic differentiation in House Sparrow populations

SHACHAR BEN COHEN & ROI DOR*

Department of Zoology, Faculty of Life Sciences, Tel Aviv University, Tel Aviv, Israel

*** Correspondence:** Roi Dor, Department of Zoology, Faculty of Life Sciences, Tel Aviv University, 69978 Tel Aviv, Israel. Email: roidor@tauex.tau.ac.il

Supplementary Tables S1-S3

Supplementary Figure S4

Supplementary Table S1:

Summary of mean (±SD) values of all morphological and color brightness traits measured in this study, by region. Traits which showed significant differences between sexes are presented for each sex separately.

		Eilat (n = 174)	Negev (n = 153)	Center (n = 139)	North (n = 226)
**Mass (g)**		24.4 ± 2.3	27.3 ± 2.7	28.5 ± 2.7	29.0 ± 3.2
**Tarsus Length (mm)**		21.4 ± 0.8	21.9 ± 0.9	21.8 ± 1.0	22.0 ± 1.2
**Bill Length (mm)**		12.4 ± 0.8	13.0 ± 0.8	12.8 ± 0.7	13.0 ± 0.8
**Wing Length (mm)**	**Males**	76.8 ± 2.3	79.4 ± 2.1	80.4 ± 2.5	80.4 ± 1.9
	**Females**	74.6 ± 2.3	76.8 ± 1.7	77.5 ± 1.6	77.6 ± 2.0
**Bill Width (mm)**	**Males**	7.3 ± 0.3	7.4 ± 0.3	7.5 ± 0.3	7.4± 0.3
	**Females**	7.1 ± 0.3	7.2 ± 0.3	7.2 ± 0.2	7.2 ± 0.3
**Color Brightness (0.0-1.0 scale)**	**Right Cheek**	0.522 ± 0.06	0.458 ± 0.06	0.438 ± 0.05	0.437 ± 0.05
	**Left Cheek**	0.513 ± 0.06	0.448 ± 0.06	0.416 ± 0.05	0.436 ± 0.05
	**Belly**	0.704 ± 0.05	0.695 ± 0.04	0.651 ± 0.05	0.631 ± 0.06

Supplementary Table S2:

Statistics for morphological traits which differed between sexes.

	**Males**	**Females**
**ANOVA**		
Wing length	$F_{1,387}$ = 180.2, P < 0.001	$F_{3,289}$ = 34.5, P < 0.001
Bill Width	$F_{3,236}$ = 3.5, P = 0. 1	$F_{3,184}$ = 0.6, P = 1
**Regression**		
Wing length	$F_{1,386}$ = 180.2, r^2 = 0.316, P < 0.001	$F_{1,291}$ = 74.3, r^2 = 0.2, P < 0.001
Bill Width	$F_{1,238}$ = 9.2, r^2 = 0.03, P < 0.01	$F_{1,186}$= 0.8, r^2 = 0, P = 0.37

Supplementary Table S3:

Summary of ten nuclear microsatellite markers used and corresponding PCR conditions.

Multi-plex	Marker	Primer sequence (5'-3')	Repeat motif	Fragment size (bp)	Annealing (35 Cycles)	$MgSO_4$ (µL)	DNA Template (µL)	Fluorescent Dye
1	**CAM01**	AAAGGCCAAG**R**CCAGTATG	CA	297-330	56°C, 1 min	3	3	6-FAM
	**CAM10**	TATCC**M**GAGAATGGGCATC	GT	173-206	56°C, 1 min	1.7	2	NED
	**CAM15**	**S**GACGACTCCTTTATTTCCC	GA	258-275	56°C, 1 min	1.7	3	ROX
	**Pdo5**	GATGTTGCAGTGACCTCTCTTG	CA	200-262	58°C, 30 sec	3.5	3	VIC
2	**CAM02**	GAATTAAAGA**Y**AGCAGATGCAGG	CA	339-357	54°C, 30 sec	2	3	ROX
	**CAM20**	TAACAGGCAGGAATGCAGG	AT	181-195	58°C, 1 min	1.6	3	NED
	**Pdo8**	ACGCCTCTGCCCAAATGAGACC	GA	162-231	66-55°C, 30 sec TD; 30 cycles 55°C.	1.8	3	6-FAM
3	**CAM05**	TTACACAGACTGCAAACCGC	CA	188-225	56°C, 1 min	1.6	3	VIC
	**CAM17**	CGGGTTGTAATCAAGAAGATGC	TG	203-208	56°C, 30 sec	3	2	ROX
	**Pdo1**	TCTGGGCTGTTGCTATCAGAAGGA	(G)6(TG)23	150-203	58°C, 30 sec	1.8	2	6-FAM

Supplementary Figure S4:

(a) An outside view of the tent in one of the sampled localities, and **(b)** a look on the arena from above as captured on the GoPro camera during an assay. Novel items are presented on the perches: **(c)** green fabric ribbon, **(d)** two soft toy balls, and **(e)** a shiny party ribbon.

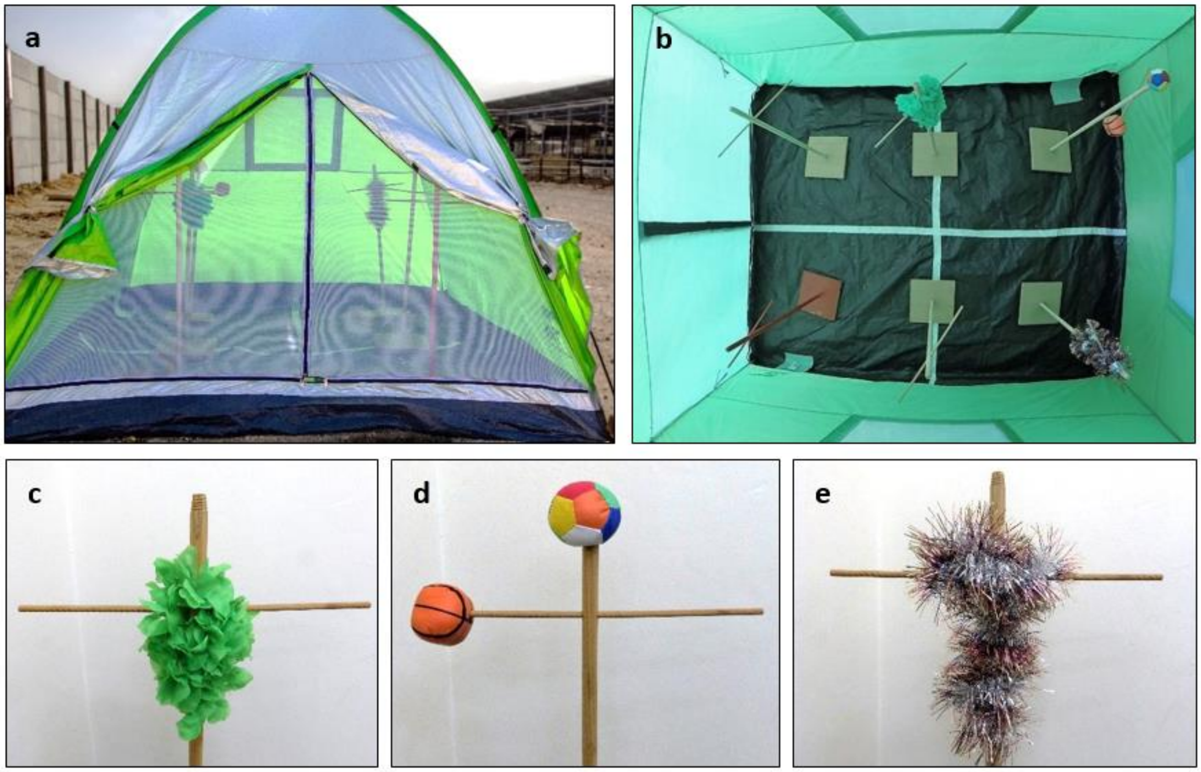

Supplement: Supplementary file 1 — Supplementary materials [file 41598_2017_18718_MOESM1_ESM.pdf]
